# Prevotella bivia Infection of a Horse Bite Wound

**DOI:** 10.7759/cureus.66571

**Published:** 2024-08-10

**Authors:** James C Barton, Alexis L Eisenberg

**Affiliations:** 1 Department of Medicine, University of Alabama at Birmingham, Birmingham, USA; 2 Southern Iron Disorders Center, Brookwood Baptist Medical Center, Birmingham, USA; 3 Department of Nursing, Veterans Administration Medical Center, Birmingham, USA

**Keywords:** prevotella bivia, oropharyngeal bacteria, horse, gram-negative bacteria, bite wound, beta-lactamase

## Abstract

Horse bites are common non-fatal injuries in the United States. Infections of horse bite wounds in humans are usually due to bacteria that correspond to the oropharyngeal bacterial flora of horses. We report the novel case of a 25-year-old woman who sustained a horse bite wound that was infected with *Prevotella bivia*, a Gram-negative, non-pigmented anaerobe. We discuss the epidemiology, bacteriology, and clinical management of horse bites.

## Introduction

Horse bites occurred in 1.7% of an estimated 102,904 people treated for non-fatal horse-related injuries yearly in 66 US hospital emergency departments during the period 2001-2003 [1]. Horse bites represented 3% of 1200 equestrian-related injuries reported in the United States in 2005 [2].

Infections of horse bite wounds in humans are usually due to bacteria that correspond to the oropharyngeal bacterial flora of horses [3]. *Prevotella *species, including *P. bivia*, are among the predominant oropharyngeal bacteria in horses with periodontal disease [4-6], although isolation of *P. bivia* from bite wounds in humans has been reported previously only in bites inflicted by dogs or cats [7] and another human [8]. We report the novel case of a 25-year-old woman who sustained a horse bite wound that was infected with *P. bivia* and discuss the epidemiology, bacteriology, and clinical management of horse bites.

## Case presentation

**Figure 1:**
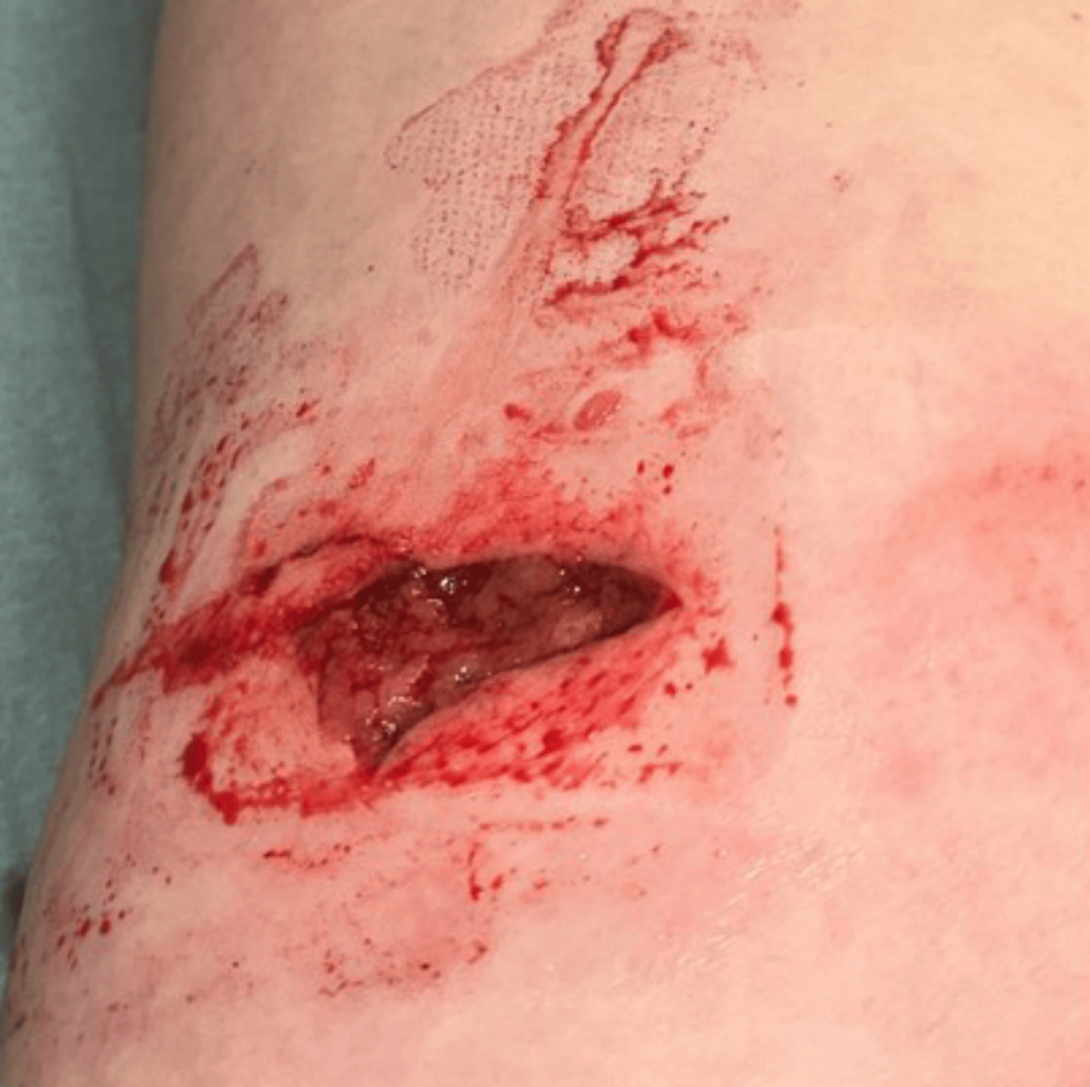
Horse bite wound of the proximal left lateral thigh. Horse bite wound of the proximal left thigh before primary debridement, cleaning, and suturing (distal, top of photograph; medial, right of photograph).

A 25-year-old woman who was riding her horse was bitten in her proximal left lateral thigh by another horse (day 0). She presented promptly to an urgent care facility. She had a history of frequent/severe sinusitis, subnormal serum IgG3 levels, and Raynaud phenomenon. She also had autoimmune hepatitis previously treated with azathioprine for five years and prednisone for six years. She took no regular prescription medications. Lobules of yellow fat were dislodged or removed by the bite (Figure 1). The wound was debrided, cleaned, and sutured. She was discharged from the urgent care facility late at night with a written prescription for amoxicillin/clavulanic acid but was unable to obtain and take amoxicillin/clavulanic acid before she developed progressive sickness.

**Figure 2:**
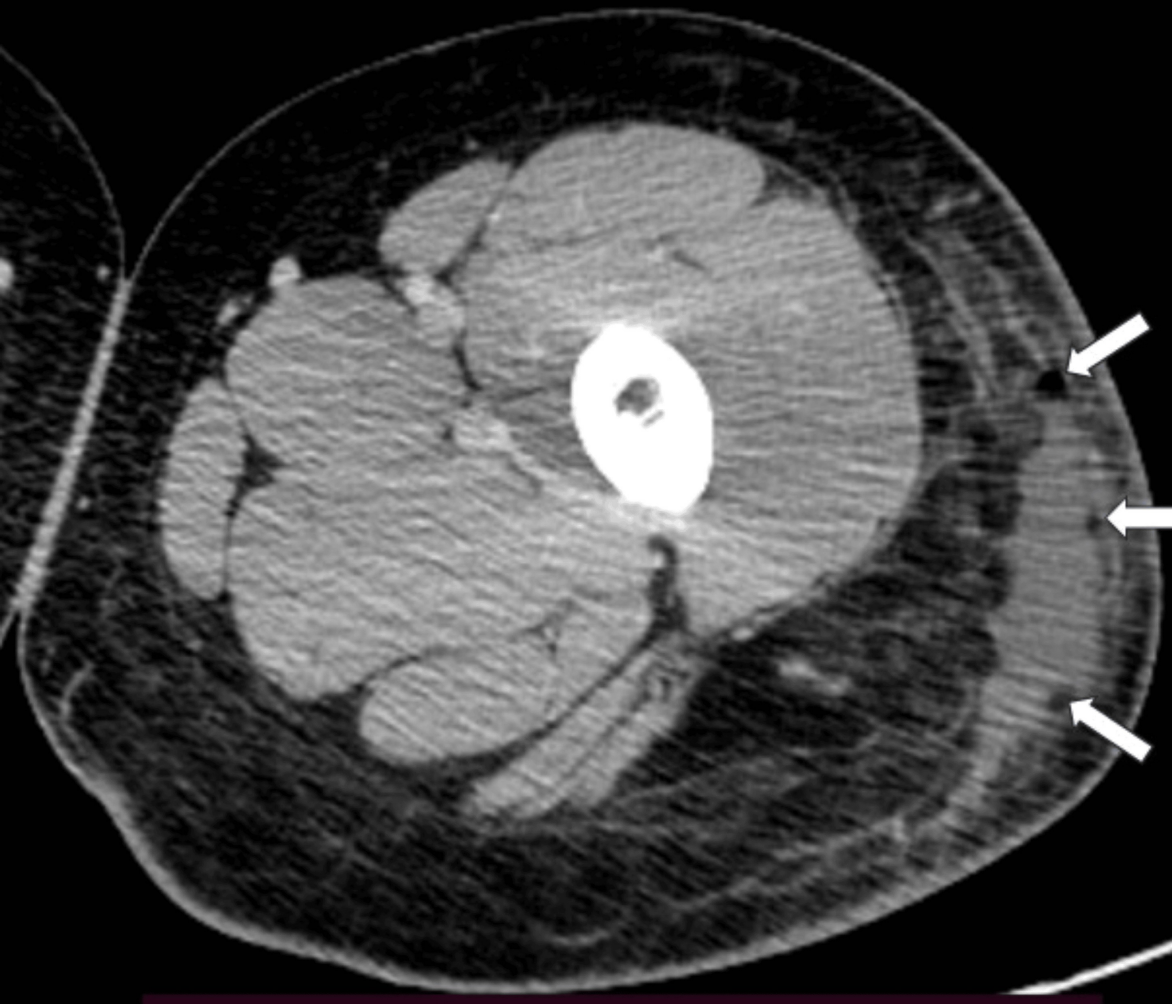
CT scan of the left thigh. CT scan with contrast imaging (2.5 mm slices) revealed an irregular crescent-shaped fluid collection and small amounts of gas (arrows) in the soft tissues of the left lateral thigh.

On day 3, she was hospitalized for presumed sepsis that emanated from the bite wound. A CT scan with contrast of the left lower extremity revealed soft tissue swelling of the lateral thigh, which was greatest in the subtrochanteric region and extended inferiorly. There was a superficial fluid collection (6.9×2.0×15.8 cm) that contained small amounts of gas (Figure 2).

**Figure 3:**
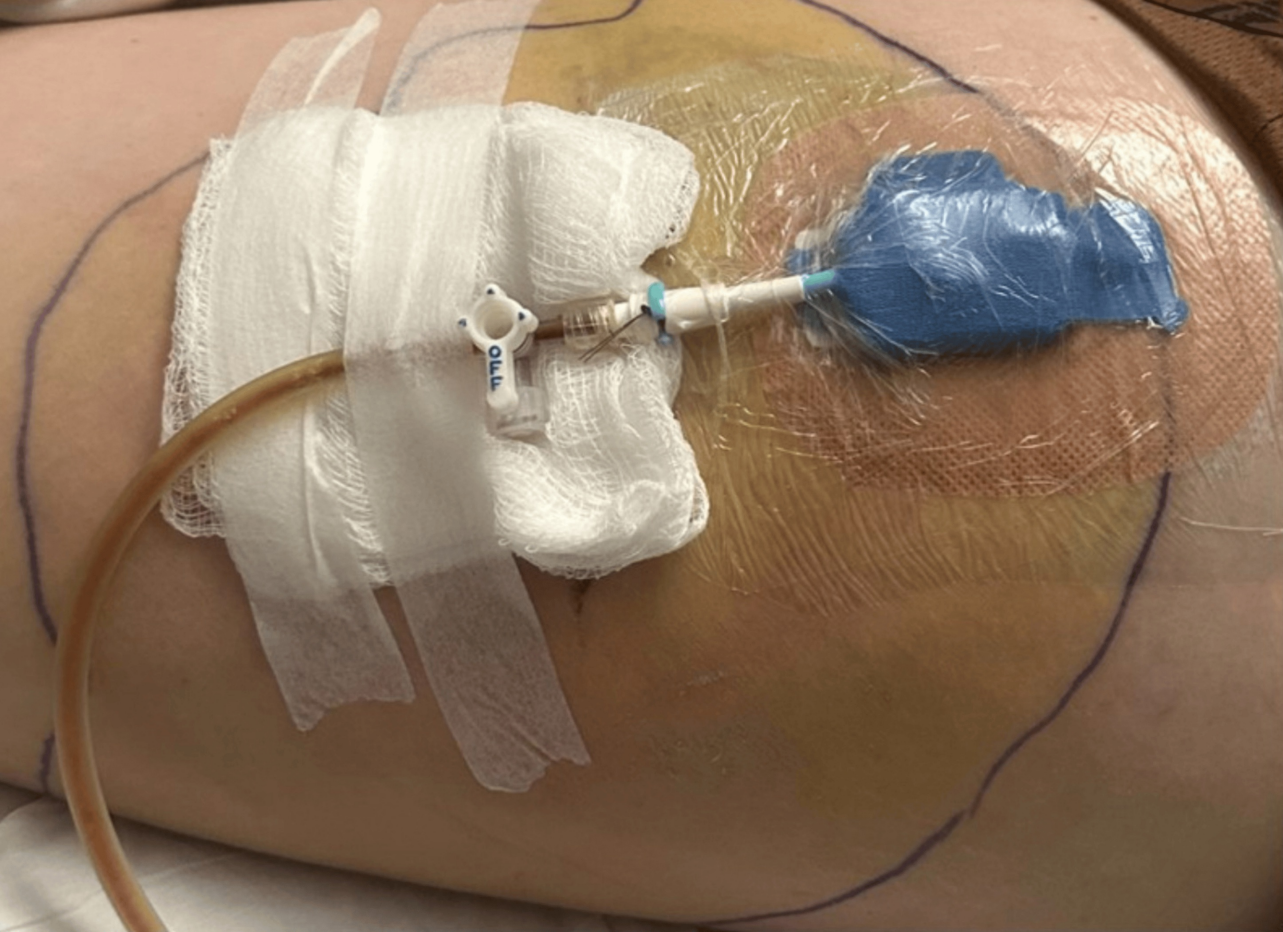
Drainage of an infected horse bite wound. Catheter drainage of a proximal left thigh abscess due to *Prevotella bivia *on day 4 after a horse bite.

She was treated empirically with intravenous piperacillin-tazobactam and vancomycin. On day 4, ultrasound-guided drainage cephalad to the bite wound produced ~300 mL of bloody fluid (Figure 3).

Blood and bite wound cultures revealed no growth during the six-day hospitalization. She was discharged from the hospital to continue wound drainage and was instructed to take doxycycline. Three days after discharge (day 12), beta-lactamase-negative *P. bivia* was identified in the bite wound culture.

She was hospitalized again during days 23-25 for progressive left thigh swelling and discomfort. A phlegmon/hematoma at the site of the bite wound was incised and drained, and she was treated empirically with a single dose of intravenous vancomycin. A Gram stain of wound fluid revealed no bacteria. Blood and wound fluid cultures revealed no bacterial growth. She was discharged with a catheter wound drain and took oral amoxicillin/clavulanic acid (875 mg/125 mg every 12 hours during days 45-58). On day 58, the drain was removed. The bite wound healed slowly although completely.

## Discussion

Rates of non‐fatal horse-related injuries in an estimated 102,904 people treated annually in 66 US hospital emergency departments during the period 2001-2003 were 1.4-fold higher in females than males [1]. Most injuries (66.1%) occurred while individuals were mounted on a horse [1]. Non‐fatal horse-related injuries occurred to the lower extremities, the upper extremities, and the head/neck regions of 22.2%, 21.5%, and 23.2% of individuals, respectively [1]. These observations are consistent with those of the present woman.

A novel aspect of the present case is the infection of a soft tissue horse bite wound with *P. bivia*, a Gram-negative, non-pigmented anaerobe. *P. bivia* in other humans was isolated from bite wounds inflicted by dogs or cats [7] and another human [8]. *P. melaninogenica* was isolated from a horse bite wound in another individual [9]. Other Gram-negative [3,9] and Gram-positive [3,10] bacteria have also been isolated from horse bite wounds in humans. We did not identify reports of *P. bivia* isolated from donkey, mule, sheep, swine, or zebra bite wounds in humans.

Nucleic acid analyses of subgingival plaque from 24 horses with periodontitis revealed a predominance of the genera *Prevotella *and *Veillonella *[5], including *P. bivia* [6], consistent with the isolation of *P. bivia* from the soft tissue horse bite wound of the present woman. We did not perform nucleic acid analyses or obtain bacterial cultures of gingival or subgingival material from the present horse. Nucleic acid analyses of gingival swabs from 24 horses with good oral health revealed a predominance of bacteria of the genera *Actinobacillus *and *Gemella *[5].

*P. bivia* also causes infections in humans unselected for reports of horse-related injuries. In association with *Gardnerella vaginalis, P. bivia* is closely linked to bacterial vaginosis [11]. *P. bivia* is a major cause of pelvic inflammatory disease [12]. Unusual *P. bivia* infections include post-COVID-19 bacteremia [13], empyema [14], and osteomyelitis of the foot of a man with diabetes [15].

The most important factor in treating animal bites is timely presentation to a medical facility or physician [16]. Emergent management of horse bite wounds recommended in a review and a case series includes cleaning with saline, debridement, and either primary closure or surgical intervention, as appropriate [16,17]. One-half of the patients with horse or donkey bite wounds in a 24-case series were satisfactorily treated with either primary closure (12 of 24 patients) or surgical intervention (12 of 24 patients) [17]. Twenty-three of the 24 patients were also treated with prophylactic amoxicillin and clavulanic acid [17]. The patient who was not treated with prophylactic amoxicillin and clavulanic acid presented with established wound infection one week after sustaining a bite wound [17]. Primary "open" wound management of horse or donkey bite wounds was not recommended [16,17].

In 500 clinical *Prevotella *isolates, not limited to those from horse bites, 59% had beta-lactamase activity, although 99.6% were sensitive in vitro to the combination of ampicillin and sulbactam, a beta-lactamase inhibitor [18]. All 188 *Prevotella *clinical isolates, including* P. bivia *not limited to those from horse bites, were susceptible in vitro to the intravenous agents piperacillin-tazobactam, cefoxitin, meropenem, imipenem, and tigecycline [19]. Both telithromycin and the combination of amoxicillin and clavulanic acid, another beta-lactamase inhibitor, were active against *P. bivia *and *P. melaninogenica* clinical isolates in vitro [20]. Prudent empiric therapy for patients with infected or probably infected horse bite wounds includes the combination of a beta-lactam antibiotic and a beta-lactamase inhibitor or a broad-spectrum lactamase-stable antibiotic [16]. Optimal specific antibiotic therapy for infected horse bite wounds varies according to bacterial isolates [16].

## Conclusions

Horse bites are common non-fatal injuries in the United States. Recommended emergent management of horse bite wounds includes cleaning with saline, debridement, and primary closure or surgical intervention, as appropriate. In one study, infections occurred infrequently in horse bite wounds treated in this manner. Despite recommended emergent management, some horse bite wounds become infected with bacteria that correspond to the oropharyngeal bacterial flora of horses. In the present woman, *P. bivia*, a Gram-negative anaerobe, was cultured from a horse bite wound. Prudent empiric therapy for patients with infected or probably infected horse bite wounds includes the combination of a beta-lactam antibiotic and a beta-lactamase inhibitor or a broad-spectrum lactamase-stable antibiotic. Optimal specific antibiotic therapy for infected horse bite wounds varies according to bacterial isolates.
